# The consequences of COVID-19 on social interactions: an online study on face covering

**DOI:** 10.1038/s41598-021-81780-w

**Published:** 2021-01-28

**Authors:** Marta Calbi, Nunzio Langiulli, Francesca Ferroni, Martina Montalti, Anna Kolesnikov, Vittorio Gallese, Maria Alessandra Umiltà

**Affiliations:** 1 Department of Medicine and Surgery, Unit of Neuroscience, University of Parma, Parma, Italy; 2 Department of Humanities, Social Sciences and Cultural Industries, University of Parma, Parma, Italy; 3 Berlin School of Mind and Brain, Humboldt-Universität zu Berlin, Berlin, Germany; 4 Department of Food and Drug, University of Parma, Parma, Italy

**Keywords:** Psychology, Human behaviour, Emotion

## Abstract

The COVID-19 pandemic has dramatically changed the nature of our social interactions. In order to understand how protective equipment and distancing measures influence the ability to comprehend others’ emotions and, thus, to effectively interact with others, we carried out an online study across the Italian population during the first pandemic peak. Participants were shown static facial expressions (Angry, Happy and Neutral) covered by a sanitary mask or by a scarf. They were asked to evaluate the expressed emotions as well as to assess the degree to which one would adopt physical and social distancing measures for each stimulus. Results demonstrate that, despite the covering of the lower-face, participants correctly recognized the facial expressions of emotions with a polarizing effect on emotional valence ratings found in females. Noticeably, while females’ ratings for physical and social distancing were driven by the emotional content of the stimuli, males were influenced by the “covered” condition. The results also show the impact of the pandemic on anxiety and fear experienced by participants. Taken together, our results offer novel insights on the impact of the COVID-19 pandemic on social interactions, providing a deeper understanding of the way people react to different kinds of protective face covering.

## Introduction

Facial expressions are extremely important for comprehending people’s emotions and intentions. Specifically, several studies have investigated facial features crucial to convey and correctly recognize specific emotions. As a result, it has been demonstrated that both the upper (particularly the eyes) and lower (particularly the mouth) face are essential for conveying and decoding emotional facial expressions. Precisely, the eyes and mouth represent crucial cues for detecting angry and happy expressions, respectively^1–7^. Previous studies have investigated the effect of covered faces on emotion perception, in particular with regard to Islamic veils or headdresses^8–10^. As expected, their results showed that when only the upper part of the face is visible (i.e., the eyes), participants perceive and recognize negative emotions (i.e., anger and fear) better than positive (i.e., happiness) ones. Furthermore, some studies also demonstrated that Islamic contextual cues bias perception toward more intense negative emotions^9,10^, showing that the interaction between contextual cues and the covering of facial features influences emotion recognition.

Recently, the COVID-19 pandemic^11^ has dramatically changed the nature of our social interactions. This is true not only of the lockdown initiated in Italy in March 2020 (when the Italian government announced restrictions on all non-essential activities, with only work and health-related travel being authorized), but also of the present days as we still regard other people as possible sources of infection. For the foreseeable future, it will be necessary to wear sanitary masks covering the lower face (i.e., mouth and nose) and do social distancing when interacting with others. How do these unprecedented conditions influence the way we perceive and comprehend other people’s emotions? Addressing this question is of utmost importance to understand the consequences of the COVID-19 pandemic on our ability to effectively entertain social interactions with others.

In the last months, many scholars have outlined the social and emotional consequences of COVID-19 on people’s well-being^12–14^, also discussing what social and behavioral science can do to support pandemic response, thus calling for a timely mobilization of the scientific community to produce research to “*directly inform individual and collective behaviour in response to the pandemic”*^15^. Some researchers responded to the call by investigating crucial aspects that can influence and promote people’ intention to adhere to distancing measures and to wear sanitary masks (e.g., content and language of messages and appeals)^16–19^. Interestingly, an online study conducted in the United States in the spring of 2020, showed that women are more prone to wear a sanitary mask than men, and that men feel more negative emotions when wearing a face covering^16^.

Few other studies mainly focused on the social negative consequences of wearing a sanitary face mask^20–23^. Indeed, the presence of the sanitary mask may influence not only the recognition and comprehension of others’ emotions, but given its current status as a contextual cue of the pandemic, also the attribution of physical and social distance. The physical distance that people maintain between themselves and others, typically defined as “interpersonal space”^24,25^, can expand or shrink depending on the situational context^24,26–28^. Indeed, it is well known that intrusions into this “personal space” induce a sense of threat and discomfort in individuals^24^. For instance, previous studies have demonstrated that individuals affected by traumatic events keep larger interpersonal distance, such as physically abused children^29^ and adults with post-traumatic stress disorder^30^.

On the other hand, it has been theorized and demonstrated that, after a traumatic collective event such as a pandemic, social distancing from members of one’s community is modulated in the opposite direction (i.e., people reduce social distancing), resulting in a renewal of the community’s sense of social bonding^31^. In other words, the common effort to fight the pandemic could augment cooperation and shared values among individuals, thus leading to the perception of being a more united and tight community with the same destiny^15^.

Another aspect to be considered is the role of virus-linked fear and stress, as they can compromise psychological resources (e.g., empathy) useful to deal with social interactions^15^.

In light of the aforementioned studies, the purpose of the present study is to understand the consequences of the COVID-19 pandemic on our ability to effectively interact with others. Specifically, the aim of the present study is fourfold: (1) to investigate how the perception of emotional facial expressions is influenced by the covering of lower-face features by different kinds of protective face-covering within the context of the COVID-19 first pandemic peak; (2) to investigate whether protective face-covering influences the attribution of physical distance as well as the perceived social distance from others; (3) to explore gender differences; (4) to investigate the current fear of COVID-19 in more detail and its relation with other personality factors.

To this aim, by means of a study available online between May 12th and June 1st 2020, Italian participants were shown static facial expressions (Angry, Happy and Neutral) in two different conditions of “covering”: by a sanitary mask and by a scarf. The scarf was chosen for its ecological validity, guaranteeing at the same time that the same portion of the face (from nose to neck) was covered. It has to be stressed that, while the sanitary mask is a personal protective equipment which ensures high protection, the scarf is not as effective as the sanitary mask, ensuring low protection from infection^32^. Participants were asked to evaluate: the emotion expressed (in terms of valence and explicit categorization), the physical distance and social distance one would adopt with the person depicted in each stimulus. With regard to emotional evaluations, we expected an impairment in the recognition of happy facial expressions as the recognition of this emotion is most affected by the covering of the lower face. We also hypothesized an increase in physical and social distance attribution to negative facial expressions. For all these aspects, a significant difference between stimuli covered with a sanitary mask and those covered with a scarf could be expected depending on the intrinsic negative value ascribed by participants to the two kinds of protective equipment, both contextual cues of the current pandemic.

## Materials and methods

### Participants

Ninety-six healthy Italian volunteers took part in the study: 47 females and 49 males, mean age 36.2 years old (Standard Deviation—*SD* = 16; min = 19, max = 82). Participants were recruited by posting announcements on social networks. The sample is quite heterogeneous, although not fully representative of the entire Italian population, with an over-representation of the age group 25–55 years old (N = 55; 57.29%) at the cost of age group > 55 years old (N = 18; 18.75%) and < 25 years old (N = 23; 23.96%). Furthermore, participants spent the COVID-19 pandemic lockdown in different regions of Italy, with an over-representation of Lombardy (30.2%), Emilia-Romagna (27.1%) and Sicily (15.6%), at the cost of other regions. For more details about the sample, please see Supplementary Materials and Supplementary Table S1 online.

The study was conducted in accordance with the Declaration of Helsinki (2013), according to the ethical standards of the Italian Board of Psychologists, as well as the Ethical Code for Psychological Research of Italian Psychological Society. As the study did not involve clinical treatments or the use of biomedical equipment with clinical implications and taking into account the necessity to run the online study promptly, approval from the local ethical committee was ruled as unnecessary. All participants provided informed consent to participate by selecting “I confirm that I have understood the terms of the present study”, thus declaring: “(i) to voluntarily adhere to the implementation of the research as a participant; (ii) to be aware that the recorded data will be analyzed exclusively for research purposes in absolute anonymity; (iii) to be aware that it is not possible to receive the recorded data back once sent”.

Power was calculated a-posteriori by means of GLIMMPSE^33^ (https://v3.glimmpse.samplesizeshop.org/#) using the Hotelling–Lawley Trace to test for a repeated-measures design with two within-factors and the Condition by Emotion interaction. The significance level was set α = 0.05 resulting in an actual power > 0.80 with our sample size.

### Procedure

**Figure 1:**
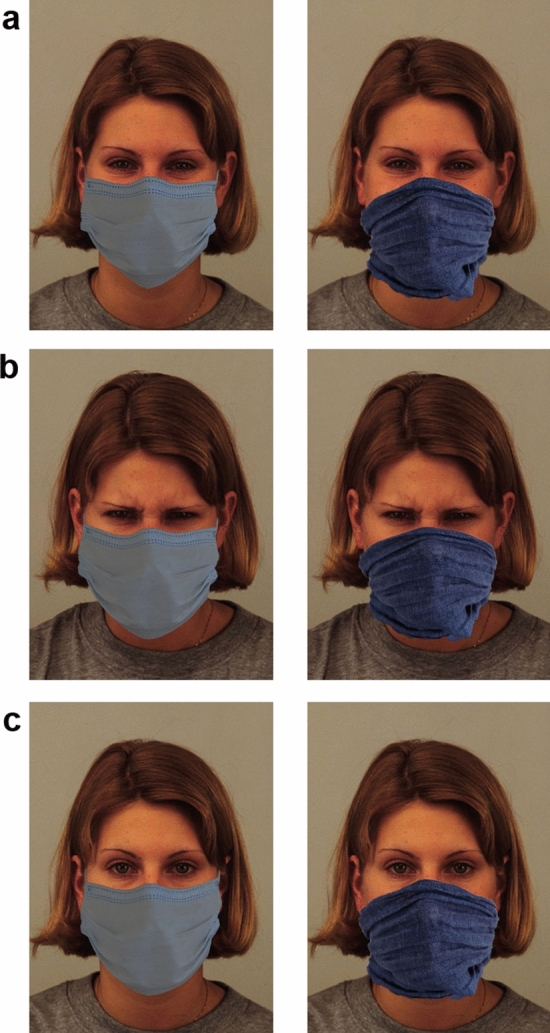
Example of stimuli. (**a**) Happiness, (**b**) Anger, (**c**) Neutral, in the two conditions of covering: sanitary mask—HP (shown on the left) and scarf—LP (shown on the right). The faces were taken from the Karolinska Directed Emotional Faces picture set—KDEF. The depicted face is AF26.

We created a study to be administered online by means of Psytoolkit^34,35^. It was composed of (1) a “socio-demographic section” divided into eight different randomized parts, each dedicated to the collection of various information (for a description see Supplementary Materials and Supplementary Tables S1–S2 online), and of (2) an experimental section dedicated to the investigation of the influence exerted by the personal protective equipment (mask and scarf) on facial expressions comprehension and on attribution of both physical and social distance. Stimuli were composed by Angry, Happy and Neutral facial expressions (6 females, 6 males) selected from the Karolinska Directed Emotional Faces—KDEF^36^. Stimuli were manipulated using Photoshop software (cc2019); specifically, a sanitary mask (from now on “High protective equipment”, HP) or scarf (from now on “Low protective equipment”, LP) was added to the faces, making sure that the same portion of the face (from nose to neck) was covered in the two conditions (see Fig. 1). Each stimulus was randomly presented four times, one for each of the following four questions: (a) Valence: “How would you judge the valence of the expressed emotion?”. Participants were asked to answer the questions using a Visual Analog Scale (VAS) from − 50 (negative) to 50 (positive); (b) Explicit categorization: “Which label would you choose to describe the person’s emotion?”. Participants were asked to choose the most appropriate emotional label in a group of seven (Anger, Happiness, Disgust, Fear, Neutral, Sadness, Surprise); (c) Social distance: The Inclusion of Other in the Self—IOS^37^ scale was used by asking: “Which picture best describes your relationship with this person (as if s/he was a member of your community)?”. Participants were asked to answer selecting one of the seven images (i.e., pair of circles) in the IOS; d) Physical distance: “How (physically) distant would you like to keep this person away from you?”. Participants were asked to answer by means of a VAS ranging from 0 (very close distance) to 100 (very high distance). Hence, this experimental section was composed of 288 “trials” in total, with the following design: 3 Emotions (Anger, Happiness, Neutral) * 12 Actors (Female, Male) * 2 Conditions (HP, LP) * 4 Questions (Valence, Explicit Categorization, Physical Distance, Social Distance). All questions were delivered in Italian. Participants were asked to observe each stimulus and to answer the question positioned below without any time constrains. Two different versions of the study were created balancing the presentation order of the two sections (socio-demographic section followed by experimental section vs. experimental section followed by socio-demographic section).

### Research design

The present behavioral experiment includes four independent variables: Emotion (3 levels: Anger, Happiness, Neutral) and Condition (2 levels: HP, LP), Participants Gender (2 levels: Male, Female) and Stimuli Gender (2 levels: Male, Female). Dependent variables are Valence, Categorization, Physical Distance and Social Distance ratings.

### Analyses

#### Valence and Physical distance

In order to investigate whether Valence and Physical Distance are modulated by the experimental conditions, a linear mixed effect analysis was performed, respectively. Following a hierarchical approach, we initially created a simple model using one parameter, and we progressively added others with the aim to evaluate whether their inclusion improved model fit. Likelihood ratio tests, Akaike Information Criterion (AIC) and Bayesan Information Criterion (BIC) were used to establish whether the inclusion of main effects, interaction effects and random effects would significantly improve model fit. We entered participants’ scores as dependent variable (Valence and Physical Distance, respectively), Emotion (3 levels: Anger, Happiness, Neutral), Condition (2 levels: HP, LP), Participants Gender (2 levels: Male, Female) and Stimuli Gender (2 levels: Male, Female) as independent fixed variables, and by participant and stimuli intercepts as random effects. Tukey’s test was used for post-hoc comparisons among means.

#### Social distance

In order to investigate whether experimental conditions modulate Social Distance evaluation, participants’ responses to the IOS scale were analyzed by means of a cumulative link model for ordinal regression using R’s clm() function^38^. Ordinal regression, selecting models by means of AIC criterion, uses a maximum likelihood estimation method within the logit model. Model convergence was assessed by inspecting the maximum absolute gradient of the log-likelihood function. The threshold was set to be equidistant from each adjacent value^38^. We considered Social Distance ratings as the dependent variable, and Emotion (3 levels: Anger, Happiness, Neutral), Condition (2 levels: HP, LP), Participants Gender (2 levels: Male, Female) and Stimuli Gender (2 levels: Male, Female) as independent fixed variables. Tukey’s test was used for post-hoc comparisons among means.

For more details about selected models please see Supplementary Table S3 online.

#### Correlations

In order to investigate the current fear of COVID-19 in more detail, we performed two Kendal correlations between the total scores for the Fear of COVID-19 scale^39,40^ with Health Anxiety^41,42^ total scores and the Interpersonal Reactivity Index^43,44^—Personal Distress (IRI–PD) subscale scores (the critical probability values for multiple comparisons were corrected with the Bonferroni method: 0.05/2 = 0.025). Health Anxiety scores were also correlated with Toronto Alexithymia Scale^45,46^ (TAS-20) total scores, TAS-20 subscales (Difficulty Describing Feelings (DDF), Difficulty Identifying Feelings (DIF) and Externally-Oriented Thinking (EOT)) scores and with IRI subscales scores (the critical probability values for multiple comparisons were corrected with the Bonferroni method: 0.05/6 = 0.008).

All analyses were performed using R software^47^ and lme4^48^, ordinal^38^, effects^49^ and emmeans^50^ packages. For data visualization we used the ggplot2 package^51^.

Analyses and results for Categorization, as well as control analyses for the effect of age on dependent measures, are presented in Supplementary Materials and Supplementary Table S4 online.

## Results

### Valence

**Figure 2:**
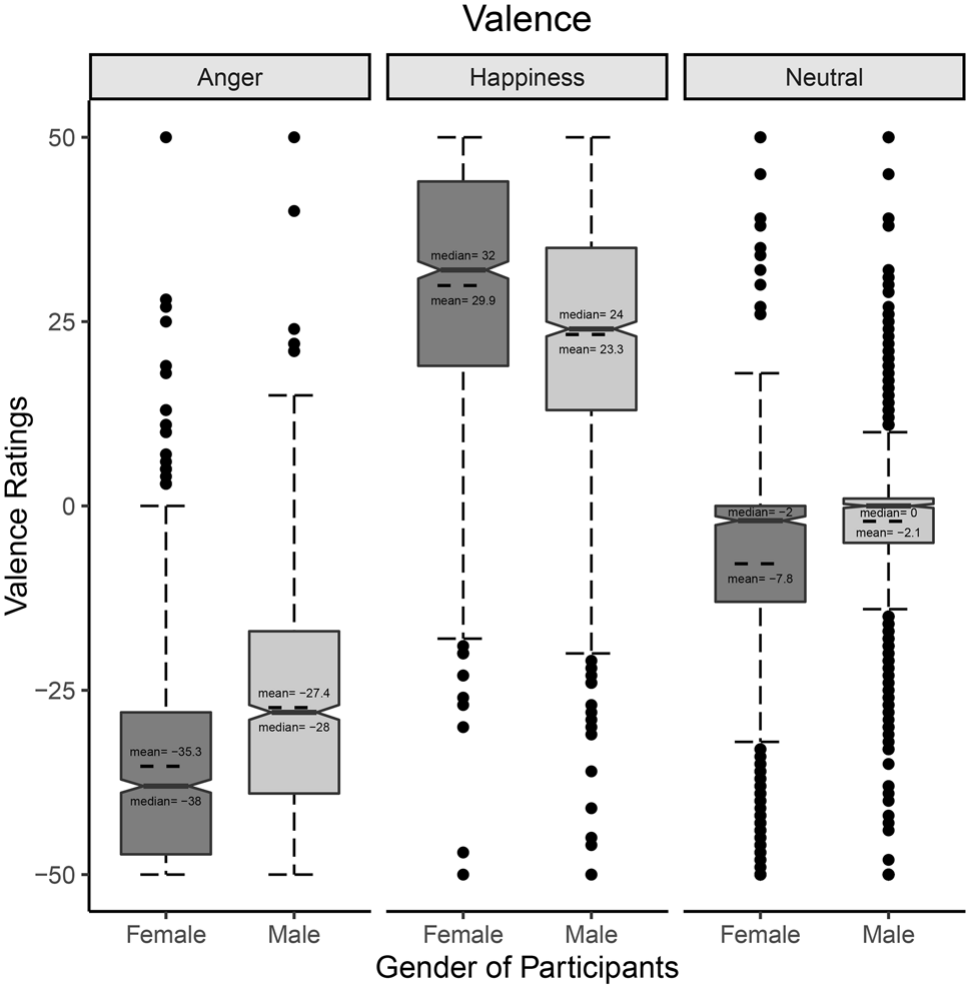
Valence: Emotion*Gender of participants’ interaction. Whisker box plot. All the differences are significant.

The model explained 76% of the variance in Valence ratings, taking into account the random effects (R^2^_m_ = 0.71; R^2^_c_ = 0.76). The model revealed a significant main effect of Emotion (χ^2^_(2)_ = 1846.5, *p* < 0.001), showing more positive ratings to Happy facial expressions than to both Angry (*z* = 43, SE = 1.35, *p* < 0.001; Happiness: M = 26.6, CIs = 24.56, 28.59; Anger: M = − 31.3, CIs = − 33.34, − 29.32) and Neutral facial expressions (*z* = 23.4, SE = 1.35, *p* < 0.001; Neutral: M = − 5, CIs = − 6.97, − 2.95). Furthermore, Valence ratings for Angry facial expressions were more negative than for Neutral ones (*z* = − 19.6, SE = 1.35, *p* < 0.001). The model also showed a significant main effect of Participants’ Gender (χ^2^_(1)_ = 7.8, *p* = 0.005): Female participants attributed more negative ratings than Male participants (*z* = − 2.79, SE = 0.84; Female: M = − 4.42, CIs = − 5.98, − 2.85, Male: M = − 2.1, CIs = − 3.61, − 0.51). Interestingly, the model also revealed a significant Emotion*Gender of participants’ interaction (χ^2^_(2)_ = 370.4, *p* < 0.001). Post-hoc tests showed that Female participants attributed more negative ratings than Males to both Angry (*z* = − 8.2, SE = 0.97, *p* < 0.001; Female-Anger: M = − 35.3, CIs = − 37.5, − 33.07; Male-Anger: M = − 27.3, CIs = − 29,6, − 25.14) and Neutral facial expressions (*z* = − 6, SE = 0.97, *p* < 0.001; Female-Neutral: M = − 7.8, CIs = − 10.1, − 5.6; Male-Neutral: M = − 2.1, CIs = − 4.3, 0.14), while they attributed more positive ratings than Male participants to Happy stimuli (*z* = 6.9, SE = 0.97, *p* < 0.001; Female-Happiness: M = 29.9, CIs = 27.7, 32.13; Male-Happiness: M = 23.3, CIs = 21, 25.47). See Fig. 2.

### Physical distance

**Figure 3:**
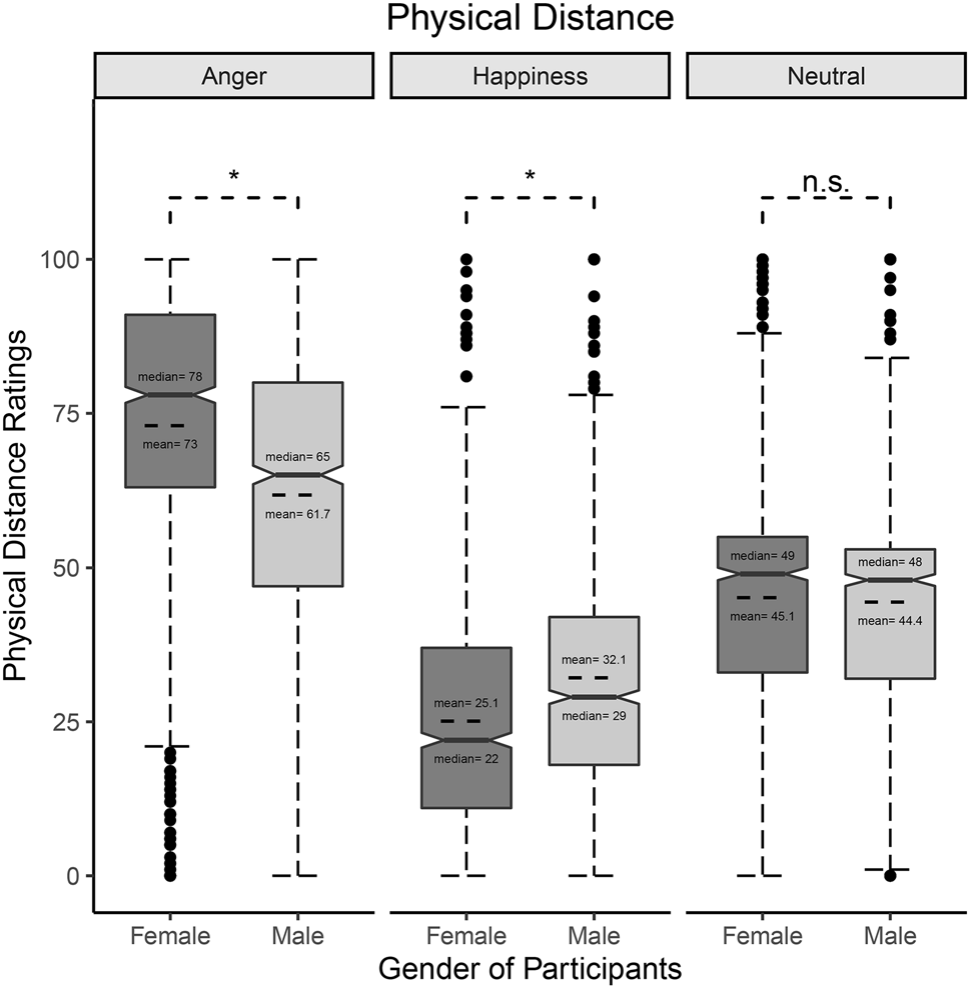
Physical Distance—Emotion*Gender of Participants’ interaction. Whisker box plot. Please note that only significant differences between Female and Male are shown for reasons of clarity. * = *p* < 0.05.

**Figure 4:**
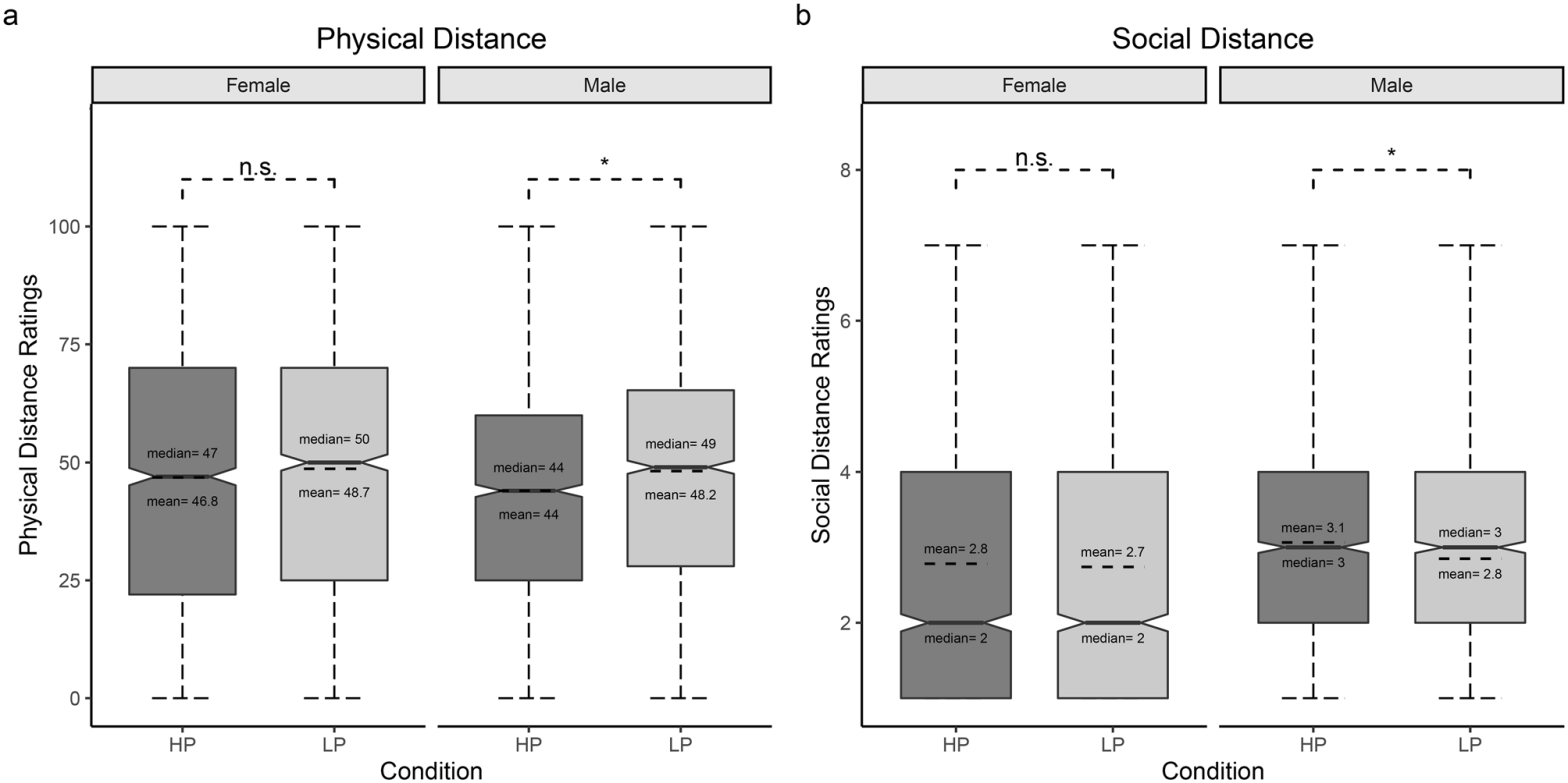
Condition*Gender of Participants interaction. Whisker box plot for (**a**) Physical Distance, and (**b**) Social Distance. Please note that in the latter, only the significant difference between HP and LP is shown for reasons of clarity. * = *p* < 0.001.

The model explained 55% of the variance in Physical Distance ratings, taking into account the random effects (R^2^_m_ = 0.37; R^2^_c_ = 0.55). The model revealed a significant main effect of Emotion (χ^2^_(2)_ = 1423.4, *p* < 0.001), showing that participants attributed greater physical distance to Angry than to both Happy (*z* = 37.7, SE = 1.03, *p* < 0.001; Anger: M = 67.4, CIs = 64.7, 70.1; Happiness: M = 28.6, CIs = 25.9, 31.3) and Neutral facial expressions (*z* = 22, SE = 1.03, *p* < 0.001; Neutral: M = 44.8, CIs = 42.1, 47.5). Furthermore, participants attributed less physical distance to Happy than to Neutral facial expressions (*z* = − 15.7, SE = 1.03, *p* < 0.001). A significant main effect of Condition was found (χ^2^_(1)_ = 13.1, *p* < 0.001), with participants attributing greater physical distance to LP stimuli with respect to HP ones (*z* = 3.6, SE = 0.84; LP: M = 48.4, CIs = 45.9, 51; HP: M = 45.4, CIs = 42.9, 48). A significant main effect of Gender of Stimuli was found (χ^2^_(1)_ = 16.3, *p* < 0.001): participants attributed more physical distance to Male stimuli (*z* = 4.03, SE = 0.84; Male stimuli: M = 48.6, CIs = 46.1, 51.2; Female stimuli: M = 45.2, CIs = 42.7, 47.8). Additionally, the model showed a significant Emotion*Gender of Participants’ interaction (χ^2^_(2)_ = 296.3, *p* < 0.001). Post-hoc tests showed that Female participants attributed more physical distance to Angry facial expressions (*z* = 4.6, SE = 2.45, *p* < 0.001; Female-Anger: M = 73, CIs = 69.4, 76.6; Male-Anger: M = 61.7, CIs = 58.2, 65.3) and less physical distance to Happy facial expressions than Male participants (*z* = − 2.9, SE = 2.45; *p* = 0.05; Female-Happiness: M = 25.1, CIs = 21.5, 28.7; Male-Happiness: M = 32.1, CIs = 28.6, 35.7), while there was no difference between males and females on physical distance ratings attributed to Neutral facial expressions (see Fig. 3). The model also showed a significant Condition*Gender of Participants’ interaction (χ^2^_(1)_ = 7.2, *p* = 0.007), but post-hoc tests did not reveal any significant difference between Male and Female participants on the physical distance attributed to stimuli covered with Mask and Scarf. Nonetheless, while Male participants attributed higher physical distance to LP than to HP (*z* = 4.4, SE = 0.94, *p* < 0.001; LP: M = 48.2, CIs = 44.7, 51.6; HP: M = 44, CIs = 40.6, 47.5), Female participants did not distinguish between the two conditions (*z* = − 1.9, SE = 0.95, *p* > 0.05). See Fig. 4a.

### Social distance

The model explained 29% of the variance (Nagelkerke pseudo.R^2^ = 0.29). The model revealed a main effect of Emotion (χ^2^_(2)_ = 7064.25, *p* < 0.001). Post-hoc tests showed that it was more likely that participants rated Angry facial expressions with lower ratings (i.e., more social distance) than both Happy (*z* = − 44.37, SE = 0.06, *p* < 0.001) and Neutral ones (*z* = − 25.24, SE = 0.05, *p* < 0.001). Furthermore, it was more likely that participants rated Happy facial expressions with higher ratings (i.e., less social distance) than Neutral facial expressions (*z* = 23.4, SE = 0.05, *p* < 0.001). The model also revealed a significant main effect of Participants Gender (χ^2^_(1)_ = 316.4, *p* < 0.001), showing that Female participants were more likely to give lower ratings than Male participants (*z* = − 8, SE = 0.04). Furthermore, the main effect of Stimuli Gender was significant (χ^2^_(1)_ = 1323.36, *p* < 0.001): it was more likely that participants rated Male stimuli with lower ratings than Female stimuli (*z* = 4.9, SE = 0.04). A significant Condition*Gender of Participants’ interaction was also found (χ^2^_(1)_ = 48.8, *p* < 0.001). Post-hoc test showed that Male participants were more likely to choose higher ratings for facial expressions covered with HP than for those covered with a LP (*z* = 4.7, SE = 0.06, *p* < 0.001), while Female participants did not distinguish between the two conditions (*z* = 1.1, SE = 0.06, *p* > 0.05). Furthermore, Female participants were more likely to choose lower ratings than Male participants (Mask: *z* = − 7.39, SE = 0.06; Scarf: *z* = − 3.93, SE = 0.06, *Ps* < 0.001) (See Fig. 4b). The model also showed a significant Emotion* Gender of Participants’ interaction (χ^2^_(2)_ = 112.02, *p* < 0.001). Post-hoc tests revealed that Female participants were more likely to choose lower ratings than Male participants for both Angry and Neutral facial expressions (Anger: *z* = − 9.5, SE = 0.08; Neutral: *z* = − 4.4, SE = 0.07, *Ps* < 0.001).

### Correlations

**Figure 5:**
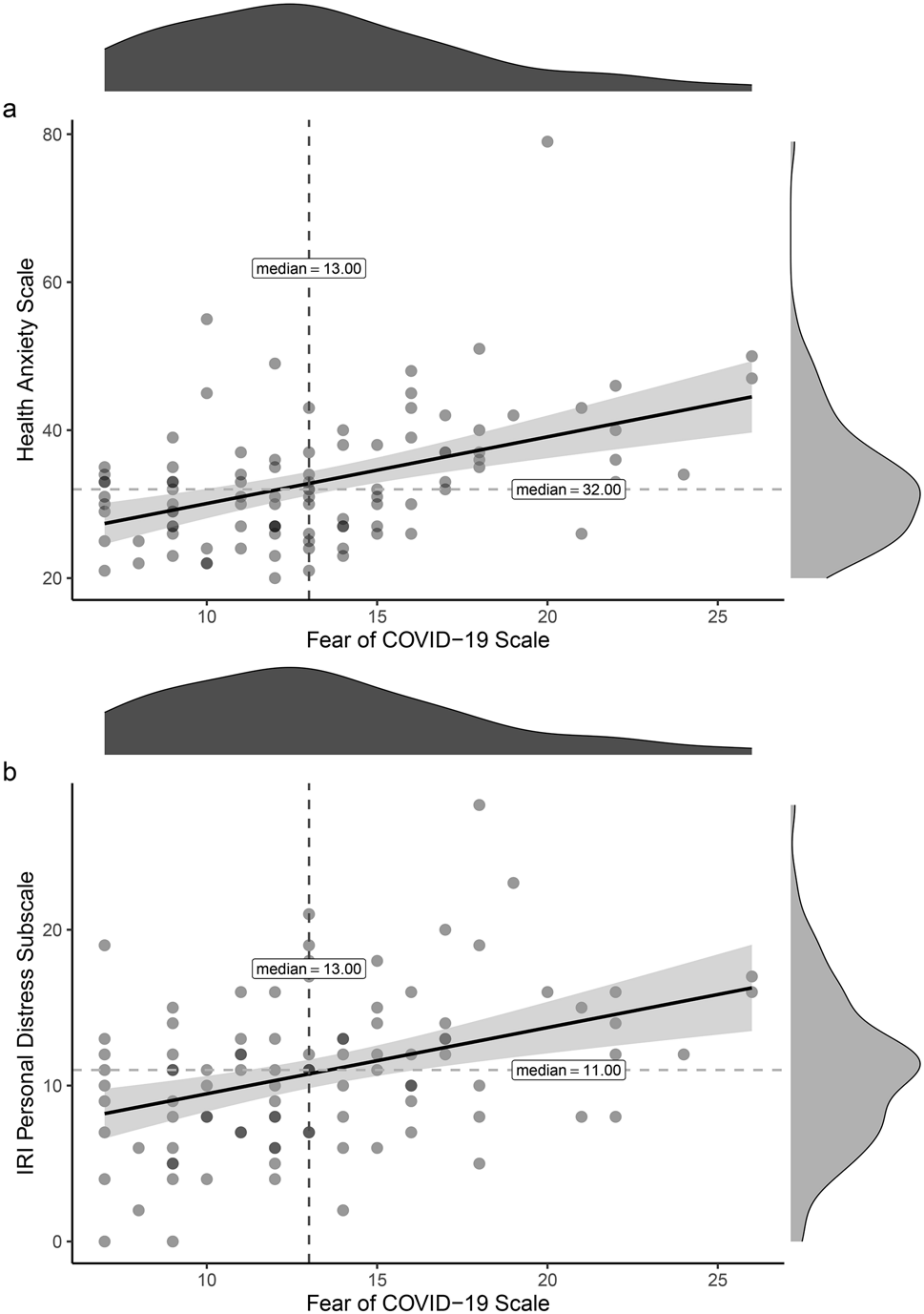
Correlations. Significant correlations between Fear of COVID-19 scale and (**a**) Health Anxiety scale, (**b**) IRI-Personal Distress subscale. Grey shadow indicates confidence intervals. * = *p* < 0.001.

The Fear of COVID-19 scale correlated with both Health anxiety (*tau* = 0.30, CIs = 0.18, 0.42; *p* < 0.001) and IRI-PD (*tau* = 0.28, CIs = 0.16, 0.41; *p* < 0.001): the higher the ratings for Health anxiety and Personal Distress, the higher the fear of COVID-19. See Fig. 5.

Analyses revealed that Health anxiety scores correlated with TAS-20 total scores (*tau* = 0.24, CIs = 0.11, 0.39; *p* < 0.001), TAS- 20 DDF (*tau* = 0.20, CIs = 0.06, 0.33; *p* = 0.005), TAS-20 DIF (*tau* = 0.34, CIs = 0.21, 0.47; *p* = 0.005) and IRI-PD (*tau* = 0.22, CIs = 0.1, 0.35; *p* = 0.002): the higher the ratings on Alexithymia and Personal Distress scales, the higher the health anxiety scores.

## Discussion

The results of the present behavioral experiment show that for Valence ratings both Female and Male participants rated Angry facial expressions as more negative than both Happy and Neutral ones, and they rated Happy facial expressions more positively than Neutral ones. Nonetheless, Female participants gave more negative ratings than Male ones when evaluating Angry and Neutral facial expressions, and more positive ratings when evaluating Happy facial expressions (see Fig. 2). While these results show that participants rated the Valence in accord with the expressed emotion, they also reveal a polarizing effect of ratings in Females. This is consistent with previous studies demonstrating women’ stronger sensibility to face stimuli (e.g., higher ratings for experienced valence and arousal, greater attention to the eyes, better decoding of emotions through facial expressions, and greater electro-cortical responsivity)^52–56^. Remarkably, the absence of a significant interaction between Emotion and Condition suggests that the presence of a HP or a LP does not modulate emotional evaluations of this kind. One could have expected lower positive ratings for Happy facial expressions covered with a scarf, which, by guaranteeing less protection for the interacting individuals, assumes a more negative contextual value. Indeed, previous studies demonstrated that contextual cues (e.g., Islamic vs. Western headdress) can bias perception toward more intense negative emotions, making the recognition of happiness more difficult^9,10^. We could hypothesize that, apart from methodological differences with previous studies, Islamic headdresses, representing cultural and religious symbols capable to evoke in-group/out-group dynamics and stereotypes^10^, are more rooted contextual cues than HP/LP, thus biasing and affecting the perception of emotions.

Results for the explicit Categorization of expressed emotions confirm participants’ ability to correctly recognize emotional expressions, as they chose emotional labels congruent with the displayed emotions. Interestingly, for Neutral facial expressions, participants also chose the Sadness label more frequently than chance, particularly in the case of Female participants (see Supplementary Table S4 online). Altogether, both the results for Valence and Categorization show that, despite the covering of the lower-face, participants were able to properly recognize the facial expressions of emotions.

Furthermore, results are also characteristic of the empirical challenges posed by operationalizing neutral emotion, and in particular of the recurrence of “negative bias” (i.e., the attribution of negative ratings to neutral stimuli)^57^. It is also possible that participants’ psychological state due to the pandemic might have influenced their responses to neutral stimuli.

With regard to Physical Distance, the expressed emotion and the condition of “covering” both influenced the results. Indeed, participants chose to keep the least distance from positive facial expressions, followed by Neutral and Angry ones. Interestingly, Female participants chose to keep greater Physical Distance from Angry facial expressions and less Physical Distance from Happy facial expressions with respect to Male participants (see Fig. 3). These results are coherent with previous studies that demonstrate an increase in interpersonal distance when participants are exposed to Angry rather than Happy facial expressions^58,59^.

With regard to the “covered” condition, less Physical Distance, only in Male participants, was attributed to facial expressions covered by HP than to those covered by LP. This latter result can be explained by the fact that the Scarf offers far less protection from the infection with respect to a sanitary mask, facilitating a higher risk for individuals if distancing measures are not undertaken^32^. Hence, it is shown for the first time that, in a pandemic context, the use of an appropriate protective device reduces the desire to maintain Physical Distance and thus potentially improves interpersonal social relations. Additionally, in accord with previous studies, we found that less Physical Distance was attributed to Female than to Male stimuli^26,60,61^.

Results for Social Distance revealed that, similarly to Physical Distance, participants chose to keep the least distance from Happy facial expressions, followed by Neutral and Angry ones, and to Female stimuli than to Male ones. While Female participants felt more socially distant than Male for stimuli covered by both HP and LP, only Male participants chose more Social Distance from faces covered with LP. Lastly, Female participants chose more Social Distance from Angry and Neutral facial expressions than Male participants. The results of the Physical and Social Distance are substantially overlapping (see Fig. 4): Females show a greater tendency, compared to Males, to keep at distance the negative or ambiguous faces regardless of the type of protective equipment (HP or LP). This is very interesting, because the need to maintain greater distance from potentially dangerous stimuli in Females seems to be driven by the emotional content of the stimuli. Differently, Males’ ratings of Social and Physical distances depend on the type of protective equipment. Females, in order to establish social and physical distance from other individuals, use more 'empathic qualities' of faces than Males, whose responses seem to be more dependent on contextual cues.

One could also have expected a difference between Physical and Social distance ratings (i.e., increase of interpersonal/physical distance and a reduction of social distance after a traumatic event; see^29–31^), but it has to be considered the period during which our data was collected. Circulation of the study began on May 12th, 2020, two months after lockdown was initiated in Italy and the World Health Organization announced that COVID-19 would be classified as a global pandemic. Indeed, previous studies demonstrate that the practice of social sharing, which enhances social bonding among members of the same community, initiates immediately after a traumatic event and does not last for longer than three weeks^31,62^.

Given the urgent need to mitigate the psychological consequences of the pandemic^15^, we carried out an investigation of current attitudes toward COVID-19 by correlating the Fear of COVID-19 scale with the Health Anxiety total scores and IRI–Personal Distress subscale scores. Results showed that the higher the scores for Health Anxiety and IRI–Personal Distress, the greater the fear of COVID-19, thus pointing for the first time to a correlation between these personality aspects and suggesting that the fear of the virus can be linked to uneasiness and nervousness during social interactions (see Fig. 5).

### Limitations, implications and recommendations

This study has some potential limitations. Firstly, we should consider that future studies are needed to investigate whether the adoption of additional control conditions would have an impact on results. Secondly, the present study was conducted on a heterogeneous, although not fully representative (for age and regions where participants spent the lockdown period), sample of Italian people. Thirdly, as the sample was composed by Italian people only, we cannot exclude that cultural diversity in the habit of wearing sanitary masks may influence results. Finally, the long duration of the study may have affected participants’ attention throughout the experiment.

Taken together, the present results offer novel insights on the consequences of the COVID-19 pandemic on our ability to effectively interact with each other. This study also highlights the impact of the pandemic on anxiety and fear experienced by participants. Future studies are needed to investigate the social and emotional costs of wearing sanitary masks (e.g., for teacher-student and clinician-patient communication as well as for individuals that are hearing-impaired)^22,63^ and how bodily postures can compensate for the lack of visible facial features. Follow-up studies are needed in order to monitor the fear and anxiety indicators taking into account potential clinical costs and social implications.

## Data availability 

The datasets generated during and/or analysed during the current study are available in the Zenodo repository, http://doi.org/10.5281/zenodo.4269905.

## Supplementary information

Supplementary Information
