# Supplementary material for: The consequences of COVID-19 on social interactions: an online study on face covering
Source: Sci Rep. 2021 Jan 28;11:2601. doi: 10.1038/s41598-021-81780-w (PMC7844002; doi:10.1038/s41598-021-81780-w)
Supplement: Supplementary file 1 — Supplementary Information [file 41598_2021_81780_MOESM1_ESM.pdf]

Supplementary Materials

The consequences of COVID-19 on social interactions: an online study on face covering.

Calbi, M.[1*], Langiulli, N.[1,] Ferroni, F.[1§], Montalti, M. [1§], Kolesnikov, A.[2], Gallese, V[1,3],

Umiltà, M.A[4].

[1] Department of Medicine and Surgery - Unit of Neuroscience, University of Parma, Parma, Italy

[2] Department of Humanities, Social Sciences and Cultural Industries, University of Parma, Parma, Italy

[3] Berlin School of Mind and Brain, Humboldt-Universität zu Berlin, Germany

[4] Department of Food and Drug, University of Parma, Parma, Italy

*Corresponding author

Department of Medicine and Surgery, Unit of Neuroscience, University of Parma, Parma, Italy

E-mail: marta.calbi@unipr.it

[§]These authors contributed equally

Materials and Methods

Procedure

The "socio-demographic section" of the study was a survey divided into eight different randomized parts, each dedicated to the collection of various information: 1) age, gender, education, nationality, region and province of residence, province where participant spent the lockdown, work in health-care, infection-related experience; 2) general health, perceived control, perceived risk of COVID-19 for self and loved ones; 3) media exposure; 4) the Italian version of the Fear of the COVID-19 Scale[1,2] 5) the Italian version of the Health Anxiety Questionnaire[3,4]; 6) the Italian version of the Toronto Alexithymia Scale – TAS-20[5,6] to assess emotional abilities; 7) items selected from two out of four subscales of the Italian version of Interpersonal Reactivity Index - IRI[7,8] to assess the empathic abilities (subscale Empathic Concern, EC) and the participant's feelings during interpersonal settings (subscale Personal Distress, PD); and 8) the Inclusion of Community in the Self[9] with the aim of evaluating participants' community connectedness.

In order to accomplish to ethic guidelines, we gave participants the possibility to take a break whenever they needed (without closing the online page, not to lose data). Thus, taking into account possible breaks, on average participants completed the whole study in 94 minutes (median=53 min.; *SD* = 188)

Table S1. Social and demographic information of participants.

**Gender**	**N**	**%**
Female	47	48.96
Male	49	51.04
Other	-	
Prefer not to tell	-	
**Highest Education**		
Middle school degree	2	2.08
High school degree	30	31.25
Bachelor degree	21	21.87
Master degree	20	20.83
Post Lauream	23	23.96
**Work condition**		
In presence/Office	12	12.50
Working remotely	44	45.83
Redundancy fund	2	2.08
Private practice	3	3.12

Unemployed	25	26.04
Retirement	10	10.42
**Subject at risk**		
Yes	18	18.75
No	78	81.25
**Loved ones at risk**		
Yes	51	53.12
No	45	46.88
**Health state**		
Absolutely bad	-	-
Pretty bad	4	4.17
Nor bad nor good	14	14.58
Pretty good	56	58.33
Absolutely good	22	22.92
**Duration of quarantine**		
< one month	13	13.54
1-2 months	22	22.92
> 2 months	61	63.54
**Use of personal protective equipment in public**		
Yes	95	98.96
No	1	1.04
**Cohabitant during quarantine**		
Alone	11	11.46
Relatives	51	53.12
Partner	31	32.29
Roommates/Friends	3	3.13
**Look up extra info about pandemic**		
Yes	80	83.33

No	16	16.67
**Which sources**		
Regular newspapers/websites/TV news	62	64.58
Social media	26	27.08
Professional websites	56	58.33
Friends and family	13	13.54
Online searches	48	50
Other	8	8.33
**Healthcare workers**		
Yes	9	9.37
No	87	90.62
**Regions of Italy**		
Lombardy	29	30.2
Emilia-Romagna	26	27.08
Sicily	15	15.62
Piedmont	6	6.25
Calabria	5	5.21
Marche	4	4.17
Tuscany	3	3.12
Lazio	2	2.08
Veneto	2	2.08
Abruzzo	1	1.04
Basilicata	1	1.04
Liguria	1	1.04
Tentino Alto-Adige	1	1.04

Almost 81% of participants did not contract COVID-19, and neither did any of their loved ones.

Table S2. Descriptive statistics for measurement as a function of questionnaire.
M and *SD* represent mean and standard deviation, respectively.

Questionnaire	***M***	***SD***
Fear of the COVID-19	13.39	4.53
Healthy Anxiety Questionnaire	33.16	8.93
TAS 20_total score	43.04	13.41
TAS 20_ subscale 1_describe	12.46	5.22
TAS 20_ subscale 2_identify	15.14	6.10
TAS 20_ subscale 3_thinking	15.45	5.34
IRI_EC	19.77	4.65
IRI_PD	10.87	4.95

Table S3 Detailed description of nested models for each measure.

Valence								
lme4::lmer(Valence ~ Emotion + Condition + Participants' Gender + Emo: Participants' Gender + Stimuli'Gender:Emo + (1|Participants) + (1|Stimuli))								
	df	**AIC**	**BIC**	**R^2_m**	**R^2_c**	**Log-Lik**	**χ^2**	***p* value**
**Reduced model (Null Model)**	2	65770.14	65783.82	-	-	-32884	-	-
**Emotion**	4	57405.01	57432.37	0.70	-	-28698	8369.1	*p* <.001
**Emotion+Condition**	5	57400.87	57435.07	0.70	-	-28695	6.14	*p* =.01
**+Participants'Gender**	6	57362.12	57403.17	0.70	-	-28675	40.75	*p* <.001
**+Emo: Participants'Gender**	8	57055.90	57110.63	0.72	-	-28520	310.2	*p* <.001
**+Stimuli'Gender:Emo**	11	57043.72	57118.97	0.72	-	-28511	18.18	*p* <.001
**+Random Intercept Participants**	12	56766.99	56849.09	0.72	0.73	-28371	278.73	*p* <.001
**+Random Intercept Stimuli**	13	56312.37	56401.30	0.71	0.76	-28143	456.63	*p* <.001

Physical Distance								
lme4::lmer(Distance ~ Emotion + Condition + Participants' Gender + Stimuli'Gender + Emo: Participants' Gender + Condition: Participants' Gender (1|Participants) + (1|Stimuli))								
	df	**AIC**	**BIC**	**R^2_m**	**R^2_c**	**Log-Lik**	**χ^2**	***p* value**
**Reduced model (Null Model)**	2	65222.1	65235.78	-	-	-32609	-	-
**Emotion**	4	62345.14	62372.50	0.34	-	-31168	2881	*p* <.001
**+Condition**	5	62314.14	62348.34	0.34	-	-31152	33	*p* <.001
**+Participants'Gender**	6	62306.32	62347.37	0.34	-	-31147	9.81	*p* =.002
**+Stimuli'Gender**	7	62267.09	62314.97	0.35	-	-31126	41.23	*p* <.001
**+Emo: Participants'Gender**	9	62064.94	62126.51	0.37	-	-31023	206.14	*p* <.001
**+Cond: Participants'Gender**	10	62061.84	62130.25	0.37	-	-31021	5.11	*p* =.02
**+Random Intercept Participants**	11	60148.84	60224.10	0.37	0.54	-30063	1915	*p* <.001
**+Random Intercept Stimuli**	12	60059.61	60141.70	0.37	0.55	-30018	91.24	*p* <.001

Social Distance							
ordinal::clm(Distance ~ Condition + Emotion + Stimuli' Gender + Participants'Gender + Emo: Participants' Gender + Condition: Participants' Gender)							
	df	**AIC**	**BIC**	**Pseudo.R^2**	**Log-Lik**	**χ^2**	***p* value**
**Reduced model (Null Model)**	2	24005.53	24019.21	-	-12001	-	-
**Condition**	3	23993.76	24014.28	0.002	-11994	13.76	*p* <.001
**+Emotion**	5	21851.04	21885.24	0.27	-10920	2146.72	*p* <.001
**+Stimuli'Gender**	6	21830.29	21871.34	0.28	-10909	22.74	*p* <.001
**+Participants'Gender**	7	21771.28	21819.17	0.29	-10879	61.01	*p* <.001
**+Emo: Participants'Gender**	9	21726.13	21787.70	0.29	-10854	49.14	*p* <.001
**+Condition: Participants'Gender**	10	21722.22	21790.63	0.29	-10851	5.91	*p* =.01

df= degrees of freedom, AIC=Akaike Information Criterion, BIC=Bayeasian Information Criterion, R^2m=marginal R^2, R^2c=conditional R2, Log-Lik = Log-Likelihood ratio test, χ^2=Chi-squared.

Analysis and results for Categorization

In order to investigate whether there was a variation in participants' choice of emotional labels across experimental conditions, we performed a Cross-Tabulation with a Pearson's chi-square test of independence.

As expected, the associations among these variables were significant ($\chi^2_{(30)} = 10373.04$, $p < .001$). Furthermore, by inspecting the individual cells in Cross-tabulation (see Table S4 below), it emerges that for Angry facial expressions, both Female and Male participants chose Anger more frequently than the other options (mean percentage = 66.8%). Specifically, the cells associated with Anger, Disgust and Fear (the latter only for Male participants) had positive adjusted standardized residual values distributed according to the Standard Normal Distribution (Mean = 0, Standard Deviation = 1, cutoff point set at |3| with $p < .01$), indicating that Female and Male participants chose these emotional labels, particularly the Anger label, significantly more than what would be expected by chance. On the contrary, cells associated with Happiness, Neutral and Sadness had negative adjusted standardized residual values, indicating that Female and Male participants chose these options significantly less than chance. For Happy facial expressions, both Male and Female participants chose the Happiness label more frequently than the other options (mean percentage = 78.6%). The cell associated with Happiness and Surprise had positive adjusted standardized residual values, indicating that Female and Male participants chose Happiness significantly more than chance, followed by Surprise. On the other hand, the cells associated with negative emotions had negative adjusted standardized residual values, indicating that Female and Male participants chose negative emotions less frequently than chance. For Neutral facial expressions, both Male and Female participants chose Neutral more frequently than the other options (mean percentage = 72.7%). Interestingly, cells associated with Sadness had positive adjusted standardized residual values, indicating that participants (females more often than males) chose this label significantly more than chance. Cells associated with all other emotional labels but Fear had negative adjusted standardized values.

Table S4. Categorization results

		Anger	Disgust	Fear	Happiness	Neutral	Sadness	Surprise
**AN – Female**	%	**71.19**	14.18	5.5	0.18	0	2.75	6.21
	χ^2	1206.5	167.3	6.8	293.3	320.5	31.5	0.1
	Adj. Std. Res.	43.1	14.5	2.9	-21.8	-23.1	-6.4	-0.3
**AN – Male**	%	**62.5**	12.76	9.78	0.26	4.17	3.49	7.06
	χ^2	850.3	122.8	101.2	304	243.3	23	0.8
	Adj. Std. Res.	36.3	12.5	11.3	-22.3	-20.2	-5.5	1
**HA – Female**	%	0	0.62	0.62	**82**	5.41	2.3	9.04
	χ^2	251.8	46.7	0.6	1324.9	210.1	38	12.4
	Adj. Std. Res.	-19.7	-7.7	-6.3	46.4	-18.7	-7	4
**HA – Male**	%	0	0.43	0.17	**75.17**	14.97	0.43	8.84
	χ^2	262.5	52.8	42.5	1062.9	74.8	75.5	11
	Adj. Std. Res.	-20.2	-8.2	-7.3	41.7	-11.2	-9.9	3.8
**NE – Female**	%	0.09	1.95	4.61	0	**67.02**	22.43	3.9
	χ^2	249.8	24	1.2	297.3	591.7	358.6	11
	Adj. Std. Res.	-19.6	-5.5	1.2	-22	31.4	21.5	-3.7
**NE – Male**	%	0.34	1.96	2.96	0.68	**78.4**	12.33	3.32
	χ^2	254.6	24.9	2.8	294.2	1034.1	41.9	17.4
	Adj. Std. Res.	-19.9	-5.6	-1.9	-21.9	41.7	7.4	-4.7

AN=Anger, HA=Happiness, NE=Neutral. Female and Male refer to gender of participants. % = percentage, χ^2= Chi-squared, Adj. Std. Res.=Adjusted Standard Residuals.

Control analyses for the effect of age on dependent measures

We performed several correlations between participants' age and the scores they gave. Specifically, for each measure (Valence, Physical Distance and Social Distance), and for each experimental condition (Anger, Happiness, Neutral; HP, LP), we performed a Kendall correlation between participants' age and their mean ratings (the critical probability values for multiple comparisons were corrected with the Bonferroni method: 0.05/5 = .01). Results showed the presence of two significant positive correlations: age correlated with both Valence scores for Anger (tau = 0.15, p <. 01) and for Neutral (tau = 0.24; p <. 001).

References

1. Ahorsu, D. K. *et al.* The Fear of COVID-19 Scale: Development and Initial Validation. *Int. J. Ment. Health Addict.* (2020) doi:10.1007/s11469-020-00270-8.
2. Soraci, P. *et al.* Validation and Psychometric Evaluation of the Italian Version of the Fear of COVID-19 Scale. *Int. J. Ment. Health Addict.* (2020) doi:10.1007/s11469-020-00277-1.
3. Lucock, M. P. & Morley, S. The health anxiety questionnaire. *Br. J. Health Psychol.* (1996) doi:10.1111/j.2044-8287.1996.tb00498.x.

4. Melli, G., Coradeschi, D. & Smurra, R. La versione italiana dell'Health Anxiety Questionnaire: Attendibilità e struttura fattoriale. *Psicoter. Cogn. e Comport.* (2007).
5. Bagby, R. M., Parker, J. D. A. & Taylor, G. J. The twenty-item Toronto Alexithymia scale—I. Item selection and cross-validation of the factor structure. *J. Psychosom. Res.* **38**, 23–32 (1994).
6. Bressi, C. *et al.* Cross validation of the factor structure of the 20-item Toronto Alexithymia Scale: An Italian multicenter study. *J. Psychosom. Res.* (1996) doi:10.1016/S0022-3999(96)00228-0.
7. Davis, M. H. Measuring individual differences in empathy: Evidence for a multidimensional approach. *J. Pers. Soc. Psychol.* (1983) doi:10.1037/0022-3514.44.1.113.
8. Albiero, P., Ingoglia, S. & Lo Coco, A. Contribute all'adattamento Italiano dell'Interpersonal Reactivity Index TT - A contribution to the Italian validation of the Interpersonal Reactivity Index. *Test. Psicometria Metodol.* (2006).
9. Mashek, D., Cannaday, L. W. & Tangney, J. P. Inclusion of community in self scale: A single-item pictorial measure of community connectedness. *J. Community Psychol.* (2007) doi:10.1002/jcop.20146.
